# The NLRP3 Inflammasome Is Upregulated in HIV-Infected Antiretroviral Therapy-Treated Individuals with Defective Immune Recovery

**DOI:** 10.3389/fimmu.2018.00214

**Published:** 2018-02-12

**Authors:** Alessandra Bandera, Michela Masetti, Massimiliano Fabbiani, Mara Biasin, Antonio Muscatello, Nicola Squillace, Mario Clerici, Andrea Gori, Daria Trabattoni

**Affiliations:** ^1^Infectious Diseases Unit, San Gerardo Hospital, Monza, Italy; ^2^Chair of Immunology, Department of Biomedical and Clinical Sciences “L. Sacco”, University of Milan, Milan, Italy; ^3^Department of Pathophysiology and Transplantation, University of Milan, Milan, Italy; ^4^Don Carlo Gnocchi Foundation ONLUS, IRCCS, Milan, Italy; ^5^University of Milano-Bicocca, Milan, Italy

**Keywords:** inflammasome, caspase, pyroptosis, HIV, immune reconstitution

## Abstract

**Background:**

Inflammasome-mediated activation of caspase-1 regulates inflammatory responses and pyroptosis. We analyzed possible associations between inflammasome-related genes and immune reconstitution in HIV-infected antiretroviral therapy (ART)-treated patients.

**Methods:**

Cross-sectional, case–control study. HIV-infected patients on ART for ≥24 months with HIV-RNA<50 cp/mL for ≥12 months were enrolled and defined as immunological responders (IR) or non-responders (INR) if CD4 count was ≥500 or ≤350 cells/μL, respectively. Expression of inflammasome genes, caspases 1, 3, 4, 5 and γ-interferon-inducible protein 16 (IFI16) was measured in unstimulated and LPS- or aldrithiol-2-treated HIV-1_BaL_ virions-stimulated peripheral blood mononuclear cells. Microbial translocation markers were evaluated.

**Results:**

Thirty-nine patients (22 IRs; 17 INRs) were enrolled. LPS-stimulated inflammasome genes were significantly upregulated in INRs. Whereas HIV-1_BaL_ stimulation induced (NOD)-like receptor (NLR) family pyrin domain containing 3 (NLRP3) expression in both IRs and INRs, NLRP3 and IL-18 expression was significantly increased in INRs compared to IRs. Significant higher caspase-1 expression was seen as well, whereas caspase 3, 4, and 5 expression was similar in both groups. No differences in microbial translocation markers (LPS and soluble CD14) were detected in the two groups.

**Conclusion:**

Upregulation of NLRP3 and caspase-1 is observed in INR patients. This could play a role in persistent immune activation that characterize INRs. Caspase-1 upregulation could induce CD4 T-cell loss *via* pyroptosis, contributing to unsatisfactory CD4 T-cells recovery.

*Summary*: Higher levels of the inflammasome genes, NLRP3 and caspase-1, are observed in ART-treated patients who lack immunological recovery. Inflammasome upregulation in immunological non-responders (INR) may be partly responsible for the persistence of immune activation and to CD4 T-cell loss *via* pyroptosis.

## Introduction

Elevated levels of inflammation and immune activation, which can persevere in HIV-infected individuals on virologically effective antiretroviral therapy (ART), have been associated to morbidity and mortality from non-AIDS events, such as cardiovascular disease, neurocognitive dysfunction, and kidney and bone disorders (1, 2). These events are more frequently observed in HIV-infected patients unable to reach a CD4+ T-cell count of 500/mm^3^, thus defined as INR.

Increases in immune activation (3) and inflammation (4) are currently believed to be the principal correlates of poor immunological response to ART, affecting T-cell pool homeostasis and modifying T lymphocytes counts at peripheral and thymic levels. Peculiar host genetic factors, as polymorphisms of genes of the pathway relative to inflammation/apoptosis (5) or genes implicated in T-cell homeostasis, such as IL7R (6), have also been correlated to suboptimal CD4+ T-cell reconstitution.

Inflammasome activation is essential for host response to pathogens, but recently clear roles for the inflammasome in the pathogenesis of inflammatory diseases has been evidenced in several studies [rev. in Ref. (7)]. The nucleotide-binding oligomerization domain (NOD)-like receptor (NLR) family, pyrin domain containing 3 (NLRP3) inflammasome is the prevalently studied among inflammasomes. Upon inflammasome activation, the NLRP3 protein recruits the inflammasome adaptor protein apoptosis-associated speck-like protein (ASC), which contains a C-terminal caspase recruitment domain, which interacts with pro-caspase-1 inducing its cleavage and activation. Inflammasome-dependent caspase-1 activity results in a specific inflammatory form of cell death known as pyroptosis, where dying cells release their cytoplasmic contents, including inflammatory cytokines, into the extracellular space (8, 9). Molecular mechanisms of chronic inflammation observed in HIV-infected patients successfully treated with ART are not clear. Recently, pyroptosis has been implicated in CD4 + T-cell loss even in absence of ongoing productive HIV replication (10–14).

As the role of the inflammasome system in the lack of immune reconstitution during ART has not been investigated till now, we analyzed possible associations between inflammasome activity, caspase-1 activation, pyroptosis, and immune reconstitution in HIV-infected ART-treated patients. Specifically, we assessed the expression of NLRP3 inflammasome, caspases 1, 3, 4, 5, and pro-inflammatory cytokines in peripheral blood mononuclear cells (PBMCs) of HIV-infected patients under suppressive ART and investigated their association with CD4+ T-cell count reconstitution.

## Materials and Methods

### Study Design

Cross-sectional, case–control study in which 39 HIV-infected ART-treated patients have been enrolled at the Unit of Infectious Diseases, San Gerardo Hospital, Monza, Italy. Patients identified as cases were HIV-infected patients defined as INR; controls were HIV-infected patients defined as immunological responders (IRs).

Inclusion criteria were: men and women >18 years of age, HIV positivity tested with ELISA and confirmed by Western Blot, treatment with combined ART (i.e., >3 antiretroviral drugs), duration of ART > 24 months, plasma HIV-RNA < 50 cp/mL for at least 12 months, CD4 + T lymphocyte count<350/mmc if INR, CD4 + T lymphocyte count >500/mmc if IRs, informed consent signed. Immunological response to ART was considered optimal when CD4+ T-cell counts were above 500 cells/mmc as these levels of immune reconstitution are associated with morbidity and mortality rates close to uninfected individuals (15).

Exclusion criteria were as follows: presence of actual opportunistic AIDS-related diseases, HCV chronic infection, chronic inflammatory disorders, and ongoing immunosuppressive therapy.

This study was conducted in accordance with the recommendations of the Ethical Committee of our Institution (Comitato Etico Brianza, Italy) including written informed consent from all patients. All subjects provided written informed consent in accordance with the Declaration of Helsinki. The protocol was approved by the Ethical Committee of Brianza on 29th May 2014.

### Blood Sample Collection and Peripheral Blood Mononuclear Cell Separation

Whole blood was collected by venipuncture in Vacutainer tubes containing EDTA (Becton Dickinson, Rutherford, NJ, USA). Plasma was stored at −80°C for further analysis and PBMCs were separated on lymphocyte separation medium (Cedarlane laboratories Limited, Hornby, ON, Canada) and washed twice in phosphate-buffered saline (PBI, Milan, Italy). Number of viable leukocytes was determined by automated cell counter ADAM-MC (Digital Bio, NanoEnTek Inc., Korea).

### Stimulation of PBMCs

Peripheral blood mononuclear cells were cultured in RPMI medium supplemented with 1% l-glutamine (Sigma-Aldrich, Milan, Italy), 1% penicillin/streptomycin (Sigma-Aldrich, Milan, Italy), and 10% Human AB Serum (Euroclone, Milan, Italy). PBMCs were incubated for 3 h either in absence or in presence of LPS (1 µg/ml) (Sigma-Aldrich, Milan, Italy) and for 24 h either in absence or in presence of aldrithiol-2 (AT2 treated)- HIV-1_BaL_ virions (300 ng/ml).

### Inflammasome Pathway Evaluation by qRT-PCR Array

Analyses were performed both in basal and stimulated conditions. mRNA was extracted from 1 × 10^6^ PBMCs by using the acid guanidinium thiocyanate–phenol–chloroform method, dissolved in RNase-free water, and purified from genomic DNA with RNase-free DNase (New England BioLabs, Ipswich, MA, USA). One microgram of RNA was reverse-transcribed into first-strand cDNA in a 20-µl final volume containing 1 µM random hexanucleotide primers, 1 µM oligo (dT), and 200 U of Moloney murine leukemia virus reverse transcriptase (Promega, Madison, WI, USA). Inflammasome signaling pathways were analyzed by a PCR array including a set of 84 optimized real-time PCR primer assays on 96-well plates (SABiosciences Corporation, Frederick, MD, USA) according to manufacturer’s instructions. The experiments have been run on samples pooled into two distinct groups (INRs and IRs) on the basis of condition (unstimulated, LPS-stimulated and AT2-treated HIV-1_BaL_ stimulated) and represent the mean value of the different targets analyzed in each group. The results were calculated relative to the housekeeping genes GAPDH and β-actin. Data were analyzed by SABioscences online software and only variables with at least a twofold increase in their value are presented, as previously described (16, 17).

### Gene Expression Analysis by qRT-PCR

For Real-Time PCR (96 CFX Connect Bio-Rad) experiments, reactions were performed using a SYBR Green PCR mix (iTaq™ Universal SYBR Green Supermix, Bio-Rad). GAPDH and β-Actin were used as housekeeping genes and the mean Ct of these two genes was used as housekeeping gene. Results are expressed as fold change 2^ΔΔCt and were calculated according to Livak’s method (18). Primers sequences were the following: GAPDH primers: forward CGGATTTGGTCGTATTGGG, reverse GCTTCCCGTTCTCAGCCTTG; β-actin primers: forward ATGCCCAGGAAGGAAGGCTG, reverse GGGAAATCGTGCGTGACATT; IL-1β primers: forward TTCTGCTTGAGAGGTGCTGATG, reverse TGTCCTGCGTGTTGAAAGATGA; IL-18 primers: forward GCTGCTGAACCAGTAGAAGAC, reverse CCGATTTCCTTGGTCAATGAAGA. For detection of NLRP3, Caspase-1, Caspase-3, Caspase-4, Caspase-5 and γ-interferon-inducible protein 16 (IFI16) primers were purchased already optimized (PrimePCR, Bio-Rad, Segrate, Italy).

### Plasma Caspase-1 Concentration

Caspase-1 was measured by Quantikine ELISA Kit (R&D Systems; Minneapolis, MN, USA) according to manufacturer’s instructions. Plasma samples were diluted 1:2 prior to assay.

### Microbial Translocation Evaluation

LPS was measured by Limulus Amebocyte Lysate (LAL) Chromogenic Endpoint Assay (Hycult Biotechnology, Uden, The Netherlands). Samples, standards, and reagents were prepared according to manufacturer’s instructions. Plasma samples were heated at 75°C for 5 min in order to neutralize endotoxin inhibiting compounds and diluted 1:5 in endotoxin free water prior to assay. Soluble CD14 (sCD14) was measured by Quantikine ELISA Kit (R&D Systems, Minneapolis, MN, USA), following manufacturer’s instructions; plasma samples were diluted 1:400 prior to assay.

### Statistical Analysis

This is a pilot study without sample size computation. Potential differences of inflammasome activation between the two groups (INR vs IR) have been evaluated.

Descriptive results have been presented as mean ± SE upon description of data distribution (median, percentiles, minimum, maximum), medians with interquartile range (IQR). Inferential statistics using either parametric (unpaired Student’s *t*-test) or non-parametric tests (Mann–Whitney *t*-test) have been used, as appropriate. Sample distributions were assessed by KS normality test. Variables associated with inflammasome gene expression were also evaluated by univariate and multivariate linear regression analysis. Data were analyzed by GraphPad Prism analysis software.

## Results

### Study Population

Thirty-nine HIV-infected patients, 17 INRs and 22 IRs, were enrolled in the study. Demographic characteristics of patients are shown in Table 1. Median age was 47 years, time of HIV infection diagnosis 10 years, time with HIV-RNA<50 cp/mL 57 months. INR patients were older (median 60 vs 43 years, *p* < 0.001) and, as expected, had a higher prevalence of past AIDS-defining illnesses (76 vs 18%, *p* < 0.001). Nadir CD4 T-cell count was significantly lower in INRs as compared to IRs [median 44 (IQR 25–83) cells/μL vs 196 (IQR 85–298) cells/μL, *p* < 0.01] Median CD4 count was 295 (IQR 256–343) cells/μL in INRs vs 840 (IQR 718–1131) cells/μL in IRs, median CD4 percentage was 19% (IQR 16–21) in INRs, and 34% (IQR 28–40) in IRs. No significant differences were observed between the two groups when years from HIV diagnosis, duration of HIV-RNA suppression, years from ART initiation and current regimen of AR were analyzed.

**Table 1:** Clinical and demographic characteristics of HIV-infected patients enrolled in the study.

	Immunological responder (*n* = 22)	Immunological non-responders (*n* = 17)	*p*
Age	43 (40–48)	60 (50–66)	<*0.001*
Male gender	16 (72.7)	14 (82.4)	ns
Caucasian	19 (86.4)	14 (82.4)	ns
Risk factor			ns
Heterosexual	11 (50)	10 (58.8)	
Homosexual	5 (22.7)	4 (23.5)	
Unknown	6 (27.3)	3 (17.6)	
Years from HIV diagnosis	9.3 (6.2–20.2)	10.2 (3.8–15.6)	ns
Past AIDS-defining events	4 (18.2)	13 (76.5)	<*0.001*
CD4 nadir	196 (85–298)	44(25–83)	<0.01
CD4	840 (718–1131)	295 (256–343)	<*0.001*
CD4%	34 (28–40)	19 (16–21)	<*0.001*
CD8	1139 (781–1383)	685 (489–899)	*0.001*
CD8%	41 (36–47)	48 (37–53)	*ns*
CD4/CD8	0.81 (0.60–1.03)	0.41 (0.29–0.58)	<*0.001*
CD4/CD8 > 1	6 (27.3)	0	*0.027*
Months from last VL > 50	53.7 (33.4–67.7)	59 (25.9–84.9)	ns
Years from cART initiation	7.7 (4.8–11.2)	10.2 (3.3–13.4)	ns
Months from last regimen initiation	48.5 (18.2–60.9)	16.8 (5.5–41.2)	ns
NRTI	20 (90.9)	16 (94.1)	ns
NNRTI	12 (54.5)	7 (41.2)	ns
PI	6 (27.3)	6 (35.3)	ns
InSTI	6 (27.3)	7 (41.2)	ns
EI	0	0	ns

*Median and interquartile ranges are shown*.

*VL, viral load. cART, combinational antiretroviral therapy*.

### Modulation of the Expression of Inflammasome Genes

**Figure 1:**
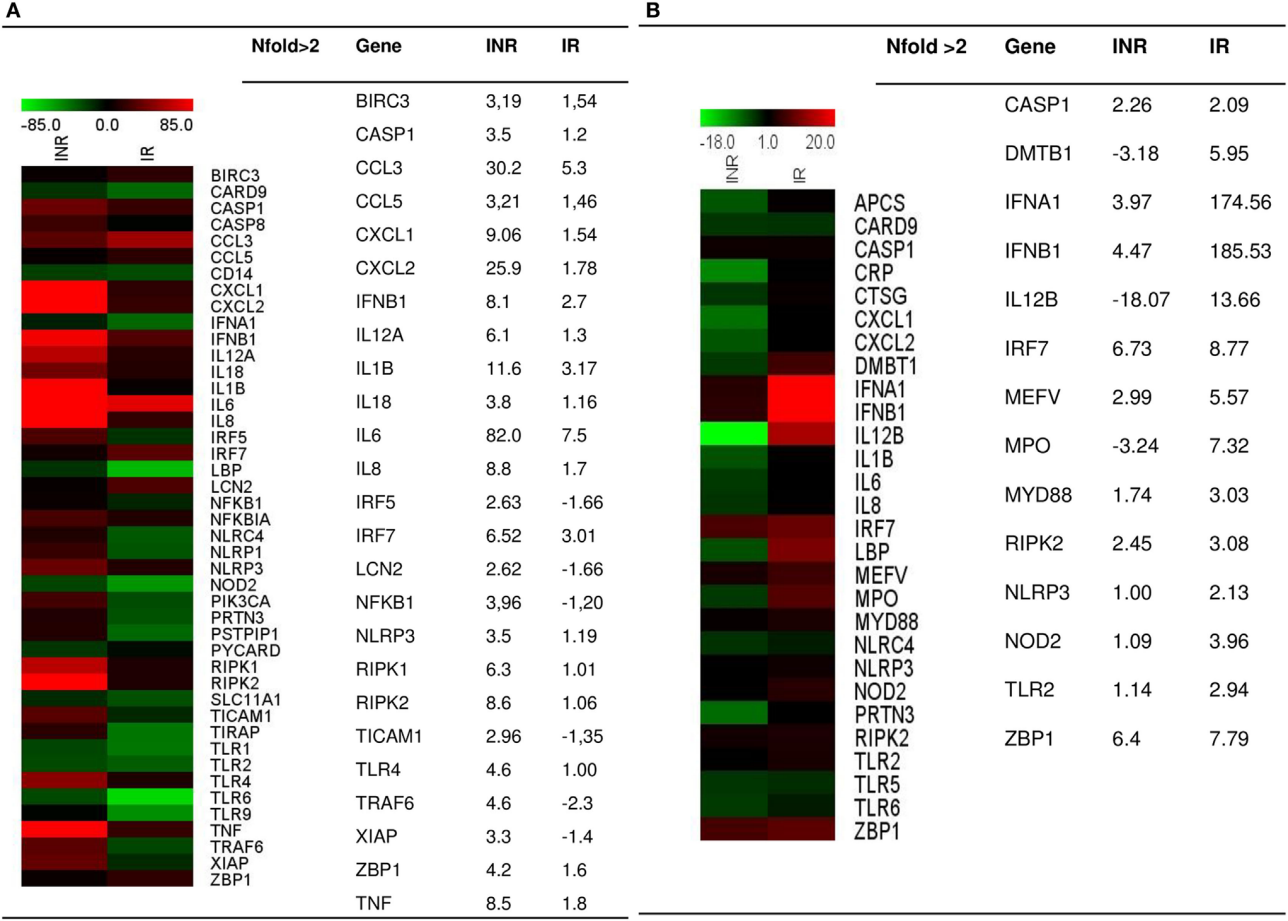
Inflammasome pathway. Real-time PCR Array on LPS- or AT2-treated-HIV-1_BaL_-stimulated peripheral blood mononuclear cells (PBMCs) in INR and IR HIV-infected patients. Samples were pooled into two distinct groups (IRs and INRs) and experiment was done in triplicate. Gene expression (*n*fold) is shown as a color scale from green to red (MEV multiple experiment viewer software). **(A)** *N*fold LPS-stimulated PBMCs vs unstimulated PBMCs; **(B)** *N*fold AT2-treated-HIV-1_BaL_ PBMCs vs unstimulated PBMCs. Only targets showing at least >2-fold modulation are shown in table.

To evaluate the expression of the inflammasome pathway, we performed a real-time PCR Array on PBMCs that were unstimulated or stimulated with either LPS or AT2-treated HIV-1_BaL_; samples were pooled into two distinct groups IRs and INRs (Figure 1). LPS is an activator of inflammasome response, whereas stimulation with AT2-treated HIV was performed in order to evaluate if the virus can potentially trigger an inflammasome response even in the context of chronic and unproductive HIV infection. Upon LPS stimulation, INRs showed an upregulation (*n*fold LPS-stimulated vs unstimulated >2) of many targets, including caspase-1, IL-1β, IL-18, NLRP3, and other pro-inflammatory cytokines, such as TNF and IL-6 and kinases involved in cellular death pathways such as RIPK1 and RIPK2 compared to IRs. AT2-HIV1-_BaL_ stimulation was less potent in triggering inflammasome responses, but we could still detect an upregulation (*n*fold AT2-HIV-1 stimulation vs unstimulated >2) for caspase-1 both in IRs and INRs, which is interesting considering its pivotal role in pyroptotic cell death and inflammation.

### Increased Inflammasome and Pyroptosis Gene Expression in INRs

**Figure 2:**
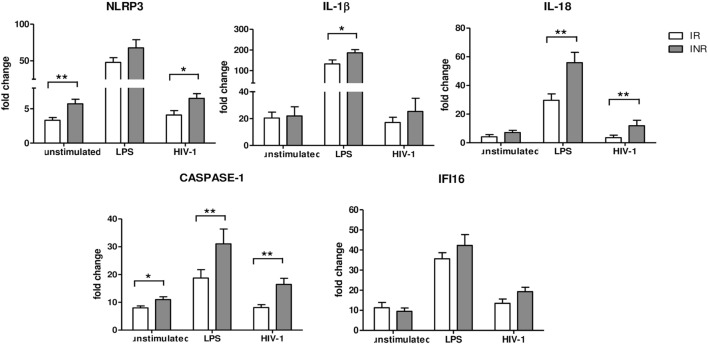
Inflammasome and pyroptosis gene expression. NLRP3, IL1β, IL-18, Caspase-1, and γ-interferon-inducible protein 16 (IFI16) expressions were quantified in peripheral blood mononuclear cells (PBMCs) from each patient enrolled in the study (*n* = 22 for IRs and *n* = 17 for INRs) either unstimulated or LPS or AT2-treated-HIV-1_BaL_ stimulated. White bars represent IRs, gray bars represent INRs. Mean values and SEM are shown. *p < 0.05; **p < 0.02. Fold change expressed as 2Δ^ΔCt^.

Given the results of the qRT-PCR array analysis, we further characterized the expression of inflammasome genes in each subject included in the study either in unstimulated or in LPS- or AT2-HIV-1_BaL_-stimulated PBMCs. In particular NLRP3, IL1β, IL-18, CASPASE-1, and IFI16 gene expression was evaluated (Figure 2). Upon LPS stimulation, we observed a significant increase of IL-1β (*p* < 0.05), IL-18, and caspase-1 (*p* < 0.02) in INRs compared to IRs. Upon AT2-HIV-1_BaL_ stimulation, we observed significant increases of NLRP3 (*p* < 0.05), IL-18 (*p* < 0.02) and caspase-1 (*p* < 0.02) in INRs compared to IRs. Of note, NLRP3 expression was significantly higher (*p* = 0.02) in INRs compared to IRs in unstimulated cells and this association was confirmed also after adjustment for either age (mean change +2.052, 95% CI 0.208–3.896, *p* = 0.03) or CD4 + T-cell nadir (mean change 2.222, 95% CI 0.277–4.167, *p* = 0.027). Altogether these data point out that a higher degree of inflammasome activation is seen in INRs compared to IRs. Of note, the gene expression of caspase-1, which is the key effector molecule of inflammasome pathway and pyroptotic cell death, was significantly higher in INRs in each condition analyzed. This association was confirmed after adjustment for CD4 + T-cells nadir value (*p* = 0.039, mean change = 15.850, 95% CI 0.860–30.839 for LPS stimulation; *p* = 0.032, mean change = 6.785, 95% CI 0.612–12.958 for AT2-stimulation) but not after adjustment for age. Expression levels of IFI16 were similar in both groups analyzed.

### Caspase-4 and -5 Are Not Involved in NLRP3 Modulation in INRs

**Figure 3:**
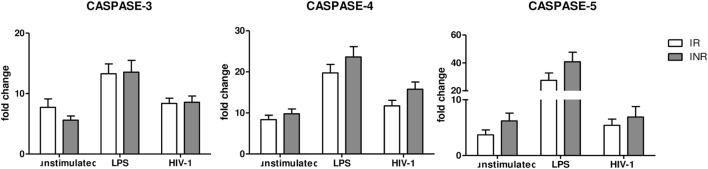
Caspase 3, 4, and 5 gene expression. Caspase 3, 4 and 5 expressions were quantified in peripheral blood mononuclear cells (PBMCs) from each patient enrolled in the study (*n* = 22 for immunological responders (IRs) and *n* = 17 for INRs) either unstimulated or LPS or AT2-treated-HIV-1_BaL_ stimulated. Mean values and SEM are shown. White bars represent IRs, gray bars represent INRs. Fold change expressed as 2Δ^ΔCt^.

NLRP3 expression can be modulated also via a “non-canonical pathway,” which is mediated by caspase-4 and caspase-5. Therefore, we decided to evaluate caspase-4 and caspase-5 gene expression in INRs and IRs as well. Results showed that the expression of these caspases was comparable in both groups of patients, indicating that the “non-canonical” pathway is not involved in NLRP3 upregulation in these patients and in the context of suppressed viremia (Figure 3).

### Apoptotic Caspase-3 Gene Expression in INRs and IRs

Caspase-3 is associated with apoptosis, a form of programmed cell death that does not lead to inflammation. We observed no significant differences between the two groups in any of the conditions analyzed (Figure 3), suggesting that caspase-3-mediated apoptosis may be similar in INRs and IRs.

### Plasma Caspase-1 Expression in INRs and IRs

We further investigated caspase-1 expression in plasma of INRs and IRs. Results indicated that higher median levels of caspase-1 expression were observed in INRs compares to IRs (INRs: 526.7 vs IRs: 375.3 pg/ml); these differences approached but did not reach statistical significance (data not shown).

### Inflammasome Upregulation Is Not Mediated by Microbial Translocation

Microbial translocation has been suggested to be the main driver of immune activation in HIV-infected patients. We hypothesized that increased expression of some inflammasomes-related genes observed in INRs could be due to increased levels of microbial translocation. To this end, we analyzed plasma levels of sCD14 and LPS, two of the main hallmarks of microbial translocation. Unexpectedly, we could not detect any differences between the two groups of patients (data not shown, sCD14 median levels: IRs 1,626,000 vs INRs 1,723,000 pg/ml; LPS median levels: IRs 46.24 vs 47.87 EU/ml), indicating that microbial translocation is not the main driver of inflammasome upregulation in INRs and that, probably, does also not play a pivotal role in immune activation in ART-treated patients.

## Discussion

Our study provides insight into one of the major mechanisms involved in the lack of immune recovery in HIV-infected subjects experiencing virological suppression under ART regimens. Differently from other studies analyzing poor immune recovery, we applied strict patients’ selection criteria, and most of the characteristics influencing immune recovery that emerged from previous studies were considered in our population (age, sex, durations of infection and treatment, co-infections, duration of HIV-RNA suppression, and nadir CD4+ counts).

Persistance of immune activation and high levels of inflammation have been identified as the underlying mechanisms inducing suboptimal immunological response to ART. We showed that NLRP3 expression is significantly increased in unstimulated or HIV-stimulated PBMCs of INRs as compared to patients who experienced complete immune recovery under ART. This result is of particular interest because, while there is ample evidence of inflammasome role during acute infection (19–22), there are no reports, to this date, on inflammasome expression during chronic infection. Importantly, we found that the condition of unsatisfactory CD4+ T-cell recovery under ART was associated with a significantly increased expression of NLRP3 even after adjustment for age and CD4+ T-cell nadir, which are known conditions able to influence immune recovery and immune activation in virologically suppressed patients. To characterize inflammasome expression in IRs and INRs, we stimulated PBMCs with LPS, which is known to be a potent activator of inflammasomes pathway. In LPS-stimulated PBMCs we observed that INRs were more responsive to LPS stimulation compared to IRs, suggesting a higher immune activation status, with an upregulation of NLRP3 and caspase-1 gene expression. In addition, we also reported a significant increase of inflammasome-related cytokines, such as IL-1β and IL-18 that are directly linked to inflammasome activation.

Next, we verified if HIV could be able to activate inflammasomes even in the settings of chronic infection as this issue has not yet been clearly addressed by other studies. Pontillo et al. reported that HIV is able to induce NLRP3 expression upon infection, but that fails to do so in chronically activated cells from infected individuals (19). Interestingly, we observed that HIV was indeed less efficient in stimulating the inflammasome pathway compared to LPS; however, we could still detect an upregulation of NLRP3, IL-1β, and IL-18 in INRs compared to IRs suggesting that inflammasome pathway is upregulated in these patients compared to IRs. Our results are apparently in contrast with pieces of evidence from a previous work by Nasi et al. who found that the expression of genes involved in inflammasome pathways did not significantly differ between HIV-infected patients under ART and healthy subjects (23). However, HIV-infected patients evaluated in that study had higher CD4+ T-cell count (about the range of normality) and inflammasome activation was analyzed only in basal conditions, whereas we found major differences after LPS- and HIV-stimulation between INR and IR patients.

Moreover, we found that INRs express significantly higher levels of caspase-1 either in unstimulated or LPS- or AT2-treated-HIV-1_BaL_ treated cells, even after adjustment for CD4+ T-cell nadir, thus suggesting a role of caspase-1-dependent pyroptosis in CD4+ T-cell death and probably in the lack of immune recovery. In the age-adjusted analysis, the association between INRs and higher levels of caspase-1 was lost; however, our analysis was partly limited by the small sample size. This finding is consistent with recent studies in which pyroptosis has been shown as a major player in CD4+ T lymphocyte loss contributing to immune activation in HIV infection. It has also been reported that this process would take place primarily in the lymph node, rather than in the blood. Indeed, even though quiescent blood CD4+ T cell support HIV entry and fusion, they are also highly resistant to pyroptotic cell death and this is probably due to their deeper state of cellular rest (13). Nevertheless, we were able to observe an upregulation of genes involved in inflammasome pathway, especially caspase-1, and this could be due to the fact that we analyzed PBMCs, which are a heterogeneous cell population compromising not only CD4+ T cells but other lymphocytes and monocytes too. Monocytes are known for constitutive caspase-1 expression (24) and release of IL-1β upon TLR4 stimulation. We can hypothesize that t the higher expression levels of inflammasome components observed in INRs could depend on increased monocytes activation in INRs compared to IRs. Of note, the array data of LPS-stimulated PBMCs, showed a significant upregulation of TLR4 in INRs compared to IRs. In agreement with reports from Muñoz-Arias et al., (13), we could not detect any differences in IFI16 expression in PBMCs from INRs and IRs, which is probably due to the resting condition of blood CD4+ T cells.

Recently microbial translocation has been considered one of the main drivers of immune activation during HIV infection and so we decided to evaluate this phenomenon in our patients. We wondered if the up-regulation of the inflammasome pathway observed in INRs could be dependent on increased levels of microbial translocation. Surprisingly, we could not detect any differences in plasma levels of LPS and sCD14 between INRs and IRs, indicating that microbial translocation is not the main driver of inflammasome and immune activation in our cohort. Consistent with our results are also recent reports by Tincati et al. and Stiksrud et al. in which no differences in LPS and sCD14 plasma levels could be detected between INRs and IRs (25, 26). It has been suggested that probably while microbial translocation markers have been shown to predict clinical outcome in untreated HIV infection, their use in ART-treated patients may not be as accurate (27).

In conclusion, it is tempting to speculate that higher inflammasome up-regulation observed in INRs may be due to residual HIV defective replication taking place in immune sanctuaries, which would lead to inflammation, immune activation and cell death. Of note, there have been reports showing that cell-to-cell transmission of HIV is not efficiently inhibited by ART and that it effectively triggers pyroptotic cell death of lymphoid-tissue-derived CD4+ T cells (11, 14), suggesting that depletion of CD4+ T cells would still take place in spite of suppressed viremia. In addition, it has been demonstrated that defective proviruses are able to transcribe novel protein-coding RNA species in HIV-infected patients on ART and with suppressed viremia (RNA levels <50 copies/ml) (28) and recent data presented at CROI 2017 demonstrated the production of HIV-1 proteins from these defective proviruses (29). These viral proteins produced even in the settings of suppressed viremia could be sensed by inflammasomes, leading to higher rates of immune activation during chronic infection and possibly to lack of immune response in INRs. The higher inflammasome upregulation observed in INRs may depend on an increased number of monocytes in these patients. It is of particular interest considering that activation of innate immunity has been linked to morbidity and mortality in the setting of ART-treated HIV infection (30, 31). Our data indicate that inflammasome and caspase-1 may be involved and play an important role in the lack of immunological response and higher immune activation status of INRs, however further investigations are necessary in order to fully prove this hypothesis. We expect that new studies on the molecular pathways involved in inflammasome, and pyroptotic cell death will shed light on molecular markers and features of these processes that could then be investigated in INRs.

## Ethics Statement

This study was carried out in accordance with the recommendations of the Ethical Committee of our Institution (Comitato Etico Brianza, Italy) with written informed consent from all subjects. All subjects gave written informed consent in accordance with the Declaration of Helsinki. The protocol was approved by the Ethical Committee of Brianza on 29th May 2014.

## Author Contributions

MM, MB, and DT performed the experiments; AB, MF, AM, and NS collected patients data and analyzed results; AB, AM, MC, AG, and DT designed the research; AB, DT, and MM wrote the paper.

## Conflict of Interest Statement

This study was supported by a grant from Gilead Fellowship Program 2013, by a grant from ANLAIDS Lombardia.
